# Co(O)_x_ Particles in Polymeric N-Doped Carbon Nanotube Applied for Photocatalytic H_2_ or Electrocatalytic O_2_ Evolution

**DOI:** 10.3390/polym11111836

**Published:** 2019-11-07

**Authors:** Xiaoxue Zheng, Ke Liu, Lichao Chen, Hengxiao He, Lusheng Chen, Chuanzhi Sun

**Affiliations:** College of Chemistry, Chemical Engineering and Materials Science, Collaborative Innovation Center of Functionalized Probes for Chemical Imaging in Universities of Shandong, Shandong Provincial Key Laboratory of Clean Production of Fine Chemicals, Institute of Materials and Clean Energy, Shandong Normal University, Jinan 250014, China; zhengxx3408@163.com (X.Z.); lrke8897@163.com (K.L.); chen04281514@163.com (L.C.); he74263664nuwy@163.com (H.H.)

**Keywords:** nanotube, photocatalytic hydrogen evolution, electrocatalytic oxygen evolution

## Abstract

A new way for synthesizing porous composite with cobalt species and N-doped carbon nanotubes (NDCNTs) was reported here by using cobalt salts and melamine mixtures as precursor. The Co(O)_x_/NDCNTs exhibited good activity of electrocatalytic O_2_ production. Furthermore, after reduced by H_2_, the Co–NDCNTs showed strong absorption of visible light and high catalytic activity of H_2_ production, which is 598.5 μmol g^−1^ h^−1^ under the visible light (λ > 420 nm). The results suggested that supramolecular preorganization of melamine monomers may be a promising method of synthesizing two-dimensional N-doped carbon nanotube with Co particles in it. The morphologies should be beneficial for the charge transport and separation. This work can encourage further synthesize new efficient noble-metal-free photocatalysts or electrocatalysts.

## 1. Introduction

With the development of economy, energy shortage and environmental pollution are becoming a serious problem. Scientists and governments are trying to find green sustainable technologies to solve this problem. Among various kinds of renewable energy, sunlight driving semiconductor photocatalyst on renewable water splitting to hydrogen fuel, has long been considered a “green strategy” to solve the limited supply of fossil fuels [1,2,3]. In the past few decades, researchers have reported varieties of photocatalysts that can be used to produce hydrogen from water [3,4]. However, although great progress and efforts have been made, there are still some challenges, for example, the low utilization of visible light and a large dependence on expensive materials and rare materials. Owing to nearly 50% of the solar energy is distributed in the visible light region, it is necessary to develop a high-efficiency visible light-driven photocatalyst without precious metals [5].

The good morphology control can effectively inhibit the photogenerated electron–hole recombination and improve the photocatalytic activity. Up to now, one effective strategy to obtain good morphology control is the template assisted processes (for example, the porous anodic aluminum oxide was used as template), which has developed the synthetic carbon nanotubes [6,7,8]. However, the steps of this method are complicated and the template needs to be removed. Another approach is to obtain preformed micron or nanostructures by using various types of organic supramolecular compounds as ordered precursors [9,10,11,12,13]. This approach is a good strategy because no template is needed. Since the specific morphological features often mean high light activity, it is important to develop strategies for controlling the morphology of catalyst.

Recently, the metal/semiconductor composite photocatalysts have attracted the attention of many researchers, which has greatly improved the photocatalytic efficiency and surpassed the single semiconductor [14,15,16]. Metal–organic supramolecular compounds self-assembled through metal coordination, hydrogen bonding and π–π interaction, are becoming a new type of porous carbon and metal oxide/carbon composite precursors [17,18,19]. Because of the highly ordered crystal structures, the metal-organic supramolecular frameworks can produce porous N-doped carbon as sacrificial templates and precursors under appropriate thermal condensation conditions. As far as we know, there are still very few reports of metal oxides/N-doped carbon nanocomposites with good control morphology through metal organic supramolecular framework.

Although metal oxide catalysts have succeeded in photochemical applications, there are still problems with most metal oxides for using only 4% of the total solar radiation. Therefore, it is very necessary to develop a catalyst with visible light response [20,21]. Many attempts have been made, for example, metal deposition (Ag [22,23], Fe [24], Zn [25], Au [26]), nonmetal doping (B [27], S [28], P [29] and F [30]), and with another semiconductor structure heterojunction composites (e.g., C_3_N_4_ hybridized ZnO [31]/Bi_2_WO_6_ [32]/TaON [33]/Ag_3_PO_4_ [34]) to improve the photocatalytic performance of photocatalysts. In the non-noble metals, cobalt-based catalysts have attracted widely attention in catalytic performance, such as, CoP [35], ZnCo_2_O_4_ [36,37] etc.

Herein, we report the synthesis of cobalt species and polymer N-doped carbon nanotube (Co(O)_x_/NDCNTs) composites with enhanced catalytic properties by using melamine and cobalt acetate as precursors. Accordingly, we develop a supramolecular arrangement method to synthesize metal (oxides) and N-doped carbon composite catalysts.

## 2. Experimental Section

### 2.1. Photocatalysts Preparation 

Co(OAc)_2_ (Sinopharm Chemical Reagent Co., Ltd., Shanghai, China) (4.9816 g, 20 mmol) and melamine (Shanghai Macklin Biochemical Co., Ltd., Shanghai, China) (2.5 g, 20 mmol) was added to methanol (Tianjin Fuyu Fine Chemical Co., Ltd., Tianjin, China) (200 mL). The mixture was refluxed for 3 h. After cooling down to room temperature, the purple solid was filtered, washed with diethyl ether (Tianjin Fuyu Fine Chemical Co., Ltd., Tianjin, China) twice and dried under vacuum. Yield: 5.0630 g (68%). 

CoO_x_–NDCNTs were fabricated by heating the above products in furnace at 550 °C for 4 h under Ar with a heating rate of 2 °C min^−1^. In addition, in order to form a comparative test, pure melamine was treated by the same method and calcined under the same conditions to obtain g-C_3_N_4_.

Co–NDCNTs were prepared by heating black powdered material of CoO_x_–NDCNTs in furnace (YKRL) at 400 °C for 2 h under hydrogen with a heating rate of 10 °C min^−1^. Then the black powdered material was treated with 1.0 M HCl (Sinopharm Chemical Reagent Co., Ltd., Shanghai, China) for 10 min, 20 min, 90 min, 24 h and 48 h. The mixture was centrifuged, and the solid material was washed three times with distilled water and dried under 60 °C. Then the resulting sample was named Co–NDCNTs-T (T represents the time of acid treatment).

### 2.2. Materials and Characterization

All chemicals were used without further purification. Powder X-ray diffraction (XRD) patterns were obtained on a Bruker D8 Advance with a graphite-monochromatized Cu Kα radiation. The morphologies of the photocatalysts were observed by transmission electron microscopy (TEM, JEM-2100, Tokyo, Japan). X-ray photoelectron spectroscopy (XPS) spectra were obtained with a PHI 5000 (Tokyo, Japan) via monochromatic Al Kα radiation. The UV–Vis absorption spectrum was obtained with X-3 spectrophotometer (Shanghai, China). The amount of hydrogen produced is detected using the GC7900 gas chromatograph (Shanghai, China) with thermal conductivity detector, 5 Å Molecular Sieve, Argon gas). Electrochemical measurements using a CHI 660D electrochemical workstation (Shanghai Chenhua Instrument Co., Ltd., Shanghai, China) with a three-electrode system with working, counter and reference electrode. Among them, the working electrode was prepared from the sample to be tested, and the counter electrode and reference electrode are Pt sheet and Ag/AgCl (saturated KCl), respectively. The electrochemical impedance spectra (EIS) and the transient photocurrent were measured using 0.1 M Na_2_SO_4_ solution as an electrolyte at a voltage 0.6 V. The working electrode was prepared by adding 2 mL of 10 μL Nafion in ethanol to 2 mg sample and dropping it onto a fluorine-doped tin oxide (FTO) glass slide (1 cm × 2 cm). The Mott–Schottky (M-S) curve was measured with an AC frequency of 1000 Hz versus −0.2 V to 1.5 V relative to a saturated calomel electrode.

### 2.3. Photocatalytic Hydrogen Production

The photocatalytic activities were evaluated by the hydrogen production under visible light irradiation (λ > 420 nm). Visible irradiation was obtained from a 300 W Xe lamp with a 420 nm cutoff filter. Hydrogen evolution was determined by online GC equipped with a thermal conductivity detector, which was connected to a closed gas-evolution system. The activity was examined in a 20 mL home-made Pyrex top-irradiation reactor at ambient temperature and atmospheric pressure. The opening of the reactor was sealed using silicone rubber septum, forming a closed system. In a typical photocatalytic experiment, 10 mg sample were placed in a quartz glass vial, an aqueous solution (10 mL) containing 20 vol% TEOA (Tianjin Fuyu Fine Chemical Co., Ltd., Tianjin, China) was added, and a vacuum was applied to remove the gas in the system and then using gas chromatograph to test the hydrogen production.

The external quantum efficiency (EQE) tests were performed with 300 W Xe arc lamp with a set of band-pass filters (=405 nm, 420 nm, 450 nm, 500 nm and 650 nm) under magnetic stirring, the irradiation intensities were determined by a NOVA II laser power meter (Ophir Photonics). The EQE was calculated by using the following equation:

(1) $$\mathrm{EQE}\;\left(\%\right)\;=\;\frac{\mathrm{number}\;\mathrm{of}\;\mathrm{reacted}\;\mathrm{electrons}}{\mathrm{number}\;\mathrm{of}\;\mathrm{incident}\;\mathrm{photons}}\;\times 100\%$$

(2) $$=\;\frac{\mathrm{number}\;\mathrm{of}\;\mathrm{evolved}\;H2\;\mathrm{molecules}\;\times 2}{\mathrm{number}\;\mathrm{of}\;\mathrm{incident}\;\mathrm{photons}\;}\;\times 100\%.$$

### 2.4. Electrocatalytic Oxygen Production

The electrolyte solution was a 1.0 M KOH (Tianjin Damao Chemical Reagent Factory, Tianjin, China) solution. The working electrode was prepared as follows: 5 mg samples were added 1 mL water-isopropanol mixed aqueous solution (volume ratio 3:1) containing 40 μL Nafion (Suzhou, China) and sonicated for 30 min to form homogeneous ink. Then 5 μL of the liquid was dropped onto a glassy carbon electrode. 

The TOF values can be calculated as follows:

TOF = j·S_geo_/4*F*·n, among them, j (mA cm^−2^) represents the current density (measured at different potentials), S_geo_ (0.07 cm^−2^) is the surface area of the glassy carbon electrode, 4 indicated that four electrons transfer are transferred to oxygen to form a carbon atom, *F* is the Faraday constant (96,485.3 C mol^−1^), and n is the number of moles of metallic cobalt. 

## 3. Results and Discussion

### 3.1. Morphology and Composition of Catalyst

**Figure 1:**
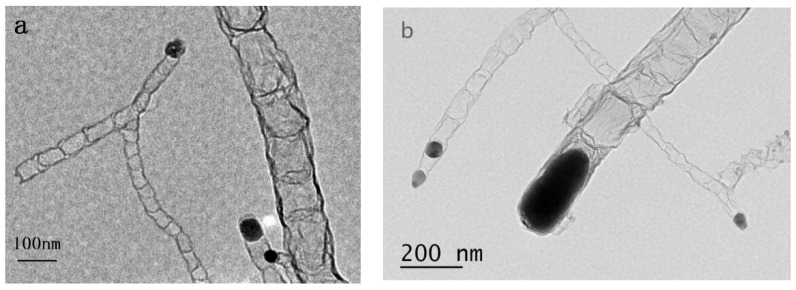
Transmission electron microscopy (TEM) images of the cobalt species and polymer of N-doped carbon nanotubes: (**a**) CoO_x_–NDCNTs and (**b**) Co–NDCNTs.

The morphologies of catalysts were analyzed by TEM CoO_x_–NDCNTs (Figure 1a) and Co–NDCNTs (Figure 1b) show nanotube morphology, as displayed in Figure 1. The TEM images indicate the presence of some carbon encapsulated cobalt nanoparticles, which is particularly at one endpoint of the nanotube. The morphologies should be beneficial for the charge transport and separation. The nanotube structure is a one-dimensional nanostructure. Usually, this kind of structure facilitates the directional transmission of electrons [38,39,40]. The TEM results suggest that the precursors obtained via the interaction between cobalt salt and melamine can polymerize to carbon nanotubes by high temperature calcination. Similar supramolecular preorganization of melamine has also been reported by Wang et al. [41] and Gao et al. [42]. The elemental analysis of different samples is shown in Table S1. It can be seen from the table that the content of N in the sample has decreased significantly after cobalt incorporation. Accordingly, the obtained CoO_x_–NDCNTs and Co–NDCNTs are the materials of N-doped carbon nanotube.

**Figure 2:**
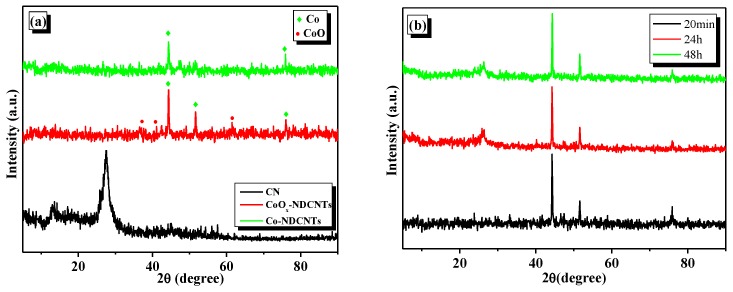
(**a**) The powder X-ray diffraction (XRD) patterns for g-C_3_N_4_, CoO_x_–NDCNTs, Co–NDCNTs and (**b**) the powder XRD patterns of different acid treatment time.

XRD pattern is shown in Figure 2. Figure 2a shows the strong peak value of 27.6°, which is the peak of g-C_3_N_4_, and was slightly higher than that of normal g-C_3_N_4_ (27.3°) [43]. It may be that the crystallinity of g-C_3_N_4_ has changed after reflux treatment. The peaks at 44.5° matched well with the characteristic peaks of crystalline Co, which shows the existence of metal cobalt in the nano-composite. In addition, the characteristic diffraction peaks of g-C_3_N_4_ cannot be observed in the composite catalyst. This may be due to the fact that part of the crystal structure of has been destroyed after thermal catalyzing by Co [44]. The peaks at 36.5°, 42.4° and 61.5° were attributed to the diffraction peaks of CoO [45]. As can be seen, after reduced by hydrogen, the peaks of CoO disappear, indicating that the CoO has been reduced to cobalt (44.2° and 75.8°). The XRD pattern clearly manifested that there are both g-C_3_N_4_ and Co in Co–NDCNTs. When Co–NDCNTs were treated in acid solution, with the increase of time, cobalt elements gradually reduced, and the peak of layered carbon gradually emerges (Figure 2b).

**Figure 3:**
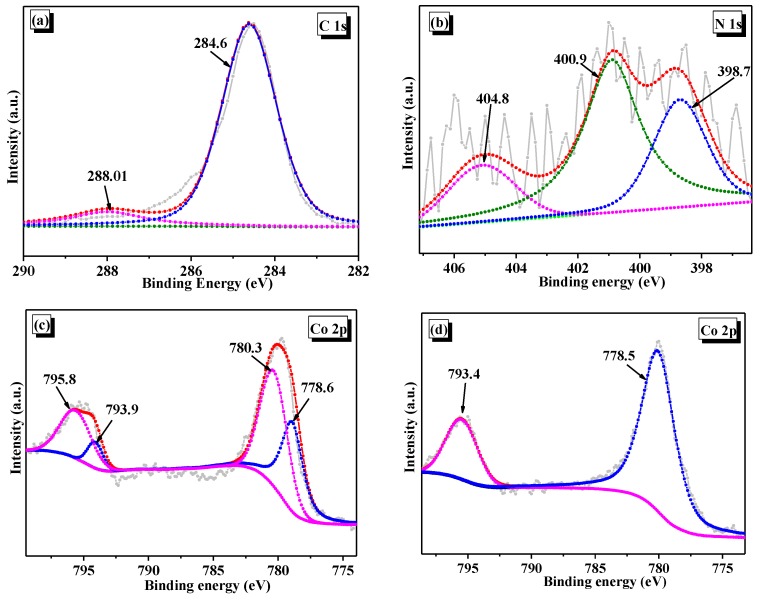
X-ray photoelectron spectroscopy (XPS) spectra for (**a**) C 1s, (**b**) N 1s, (**c**) Co 2p of CoO_x_–NDCNTs and (**d**) Co 2p of Co–NDCNTs.

The chemical components of Co–NDCNTs were analyzed by X-ray photoelectron spectroscopy (XPS), as shown in Figure 3. The high resolution C1s XPS spectra at 288.01 eV was ascribed to sp^2^-bonded carbon of N=C–N [46]. The high resolution N1s XPS spectra were divided into three peaks at 398.7, 400.9, 404.8 eV, respectively. The peak at 398.7 eV was assigned to sp^2^ hybridized aromatic N bonded to carbon atoms of C–N=C [47]. The peak at 400.9 eV was considered to be indicative of the tertiary nitrogen N–C_3_ [48]. The weakest peak at 404.8 eV is belonged to the π-excitations [49]. Figure 3c shows the Co 2p of CoO_x_–NDCNTs, with the two peaks at 793.9 eV and 778.6 eV, respectively, representing 2p_1/2_ and 2p_3/2_ of metal Co. The other two peaks (795.8 eV and 780.3 eV) are the signal of Co2p_1/2_ and Co2p_3/2_ of Co^2+^ [50,51,52]. Figure 3d shows the Co 2p of Co–NDCNTs, the two peaks at 793.4 eV and 778.5 eV are attributed to the characteristic peaks of 2p_1/2_ and 2p_3/2_ of metal cobalt, respectively. These results suggest that cobalt species has been reduced to metal cobalt after hydrogen treating, which is agreement with the XRD results.

### 3.2. Optical and Electrochemical Properties of Samples

**Figure 4:**
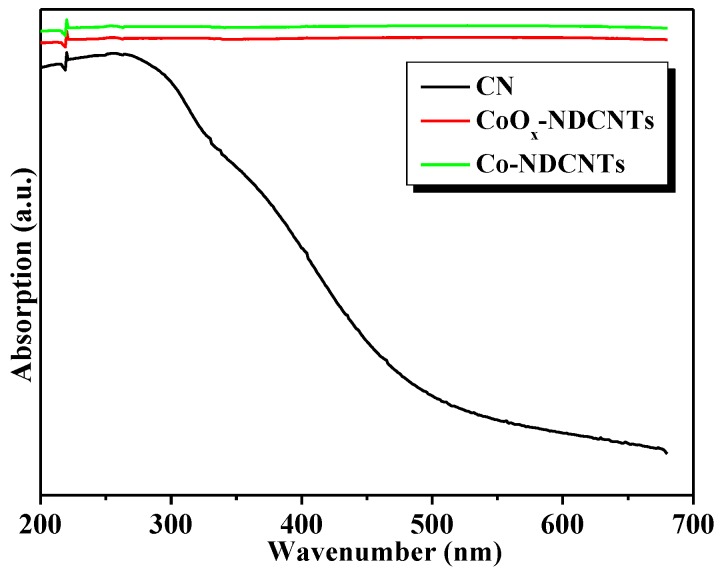
UV–Vis of pure g-C_3_N_4_, CoO_x_–NDCNTs and Co–NDCNTs.

The optical properties were studied by UV-Vis diffuse reflectance spectra (Figure 4). The absorption edge of pure g-C_3_N_4_ is at about 455 nm, which is corresponding to a band of 2.73 eV. Although CoO_x_–NDCNTs and Co–NDCNTs were also active in the visible region, its UV–Vis spectrum is a straight line because of the black color of the catalyst (Supplementary Materials, Figure S1).

**Figure 5:**
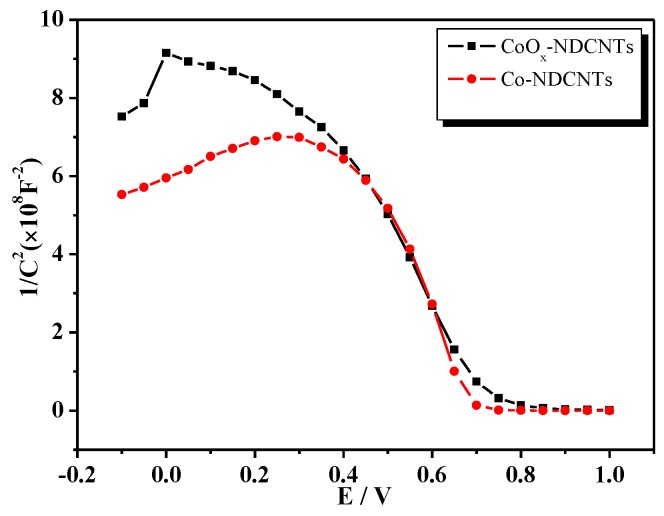
Mott–Schottky patterns of CoO_x_–NDCNTs and Co–NDCNTs samples.

The flat band potential (EFB) is an important parameter for the semiconductor/solution system. Its numerical value can be used to describe the energy level structure of semiconductor electrode. Figure 5 shows that in the experimental voltage range, the slope of the M-S curve is negative, indicating that semiconductors exhibit p-type semiconductor characteristics [53].

**Figure 6:**
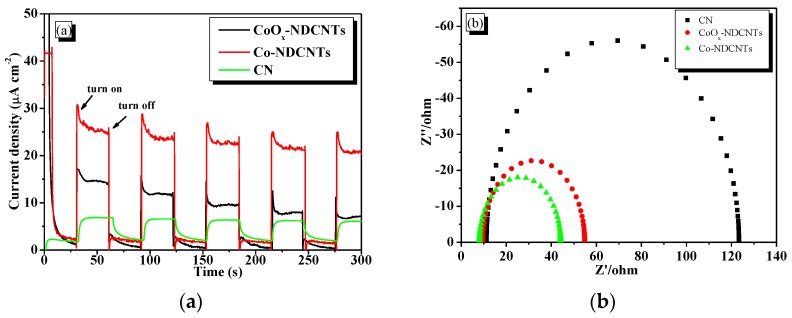
(**a**) The transient photocurrent response under visible light irradiation and (**b**) electrochemical impedance spectra (EIS) spectra of g-C_3_N_4_, CoO_x_–NDCNTs and Co–NDCNTs recorded at 0.6 V.

Photogenerated electron-hole separation and electron transfer can be studied by means of electrochemical characterization. Photocurrent responses of g-C_3_N_4_, CoO_x_–NDCNTs and Co-NDCNTs are shown in Figure 6a. It can be seen that under the excitation of visible light, both samples can quickly show a stable photocurrent response, indicating the existence of photo-generated carriers in the samples. Compared with g-C_3_N_4_, the CoO_x_–NDCNTs and Co–NDCNTs sample shows the higher photocurrent response, and the photocurrent intensity of Co–NDCNTs sample is about 3.0 times that of g-C_3_N_4_. The results show that the photogenerated electron-hole pairs in the Co-NDCNTs can be more effectively separated than those in g-C_3_N_4_. EIS was further aimed at measuring the charge transfer resistance that controls the charge transfer kinetics of the redox probe at the interface of the photocatalyst [54]. The Nyquist plots for pure g-C_3_N_4_, CoO_x_–NDCNTs and Co–NDCNTs are shown in Figure 6b. The radius of the Nyquist curve is proportional to the resistance of the electrode surface, and the smaller the radius is, the smaller the resistance of the electrode. From the Figure 6b, it can be seen that the *R*ct of CoO_x_–NDCNTs, Co–NDCNTs are much smaller than that of pure g-C_3_N_4_, demonstrating the metal Co loaded on g-C_3_N_4_ can substantially promote the charge transfer and suppress the recombination rate of photoinduced electron-hole pairs.

### 3.3. Photocatalytic Hydrogen Production Activity of Samples

**Figure 7:**
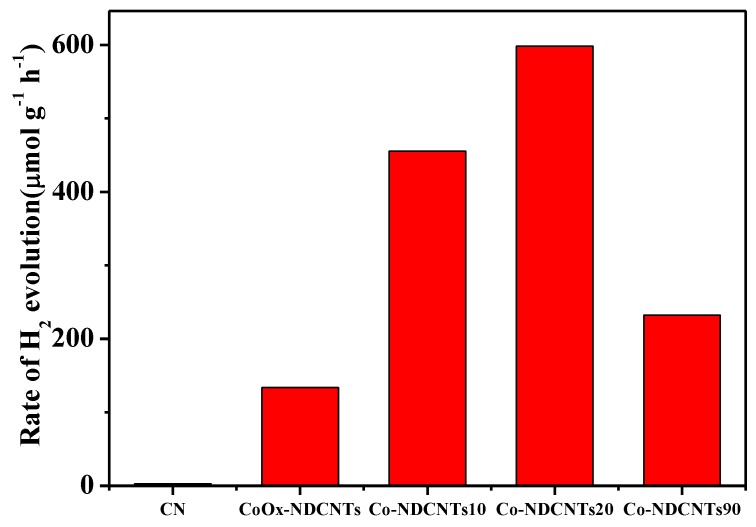
The calculation of the hydrogen generation rate of photocatalyst prepared.

As shown in Figure 7, under visible light irradiation, the rate of hydrogen production by pure g-C_3_N_4_ is almost 0, suggesting that the pure g-C_3_N_4_ itself show very low visible light activity. All composite materials have better visible light catalytic activity than that of pure g-C_3_N_4_, which further demonstrates that the addition of Co(O)_x_ effectively inhibits the recombination of photogenerated electrons and holes, increases the rate of charge transfer and improves the photocatalytic activity. With the increase of acidic treatment time, the activity of the catalyst increases firstly and then decreases. The highest amount of H_2_ is produced by the Co–NDCNTs-20 (598.5 μmol g^−1^ h^−1^) catalyst. The increase of the activity may be attributed to the optimum content of metallic cobalt as cocatalyst. However, the decrease of the activity should be due to the excessive reduction of cobalt, which has been dissolved in HCl. Therefore, the results show that the proper amount of metal cobalt is beneficial to the improvement of photocatalytic activity. In addition, as shown in Table S2, the external quantum efficiency (EQE) of Co–NDCNTs-20 was measured to be 1.83%, 1.28%, 0.78% and 0.47% at wavelengths of 405 nm, 420 nm, 450 nm and 500 nm, respectively. It can be seen that the Co–NDCNTs-20 photocatalyst shows considerable quantum efficiency when the wavelength is higher than 450 nm. 

**Scheme 1:**
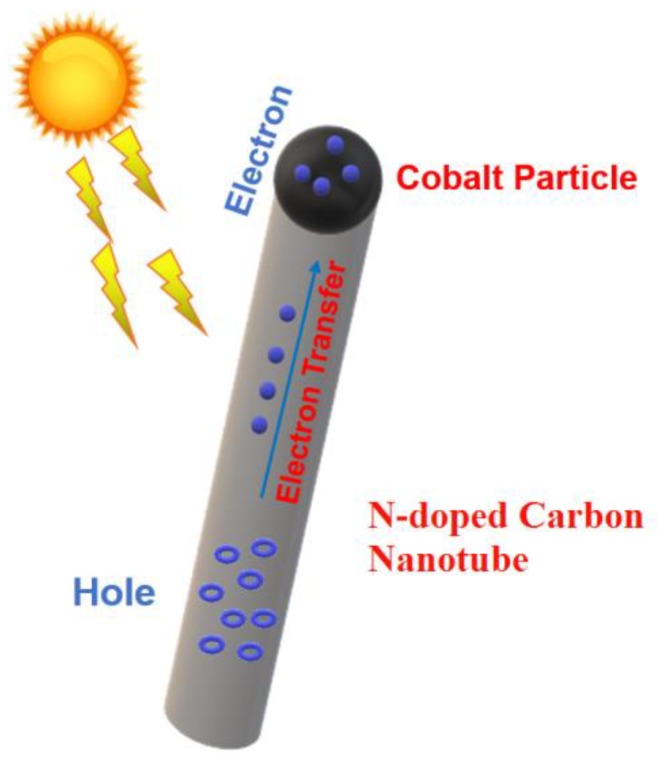
The mechanism of the photocatalytic hydrogen production under visible light irradiation.

As shown in Scheme 1, under the irradiation of visible light, electrons are excited from the valence band to the conduction band of the N-doped carbon nanotube, and holes are generated. Metals such as nickel [55] and cobalt [56] have excellent electronic conductivity. Therefore, the electrons on the conduction band are transferred to the cobalt particles, which accelerate the separation of electrons and holes in the N-doped carbon nanotube, thereby improving the photocatalytic hydrogen production activity. Zhao et al. [57] previously reported similar catalytic mechanism of tubular structures.

### 3.4. Electrocatalytic Oxygen Production Activity of Samples

**Figure 8:**
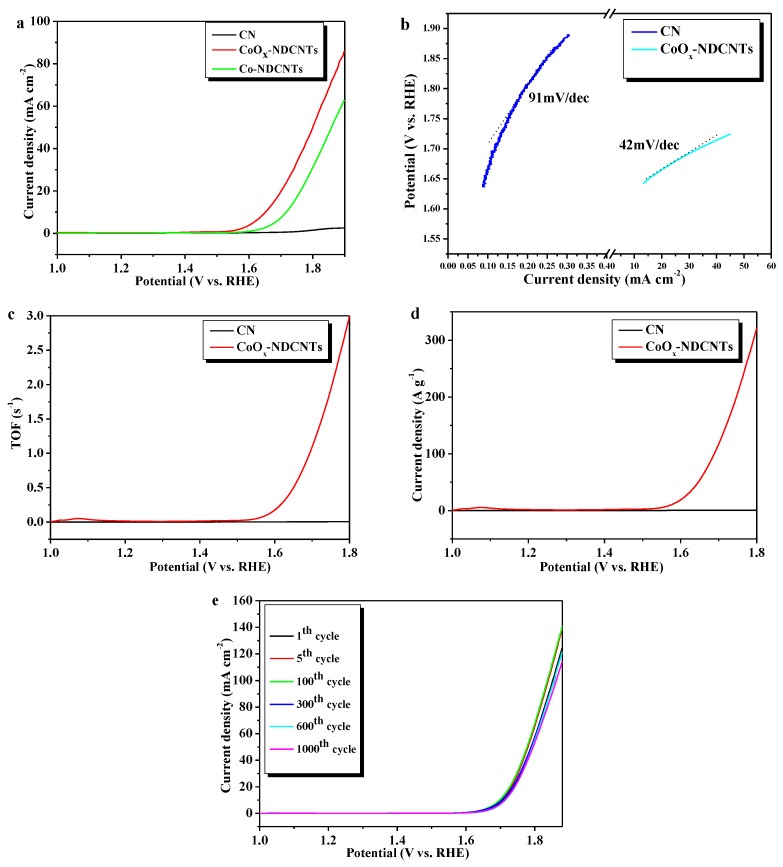
Electrochemical characterizations on oxygen evolution reaction (OER) activity (**a**) polarization curves, (**b**) tafel plots in 1 M KOH medium, (**c**) TOF plots with respect to applied potentials in 1M KOH electrolyte, (**d**) mass activity of diverse OER catalysts and (**e**) stability test of the CoO_x_-NDCNTs nanotube by long-term CV cycling.

Electrochemical measurements were performed to verify the structural advantages of CoO_x_–based nanotube catalyst in a typical three-electrode configuration in 1 M KOH. As shown in Figure 8a, the polarization curve recorded with the CoO_x_–NDCNTs nanotube reveals a markedly small onset potential of 1.54 V for the oxygen evolution reaction (OER). In contrast, the pure g-C_3_N_4_ and Co–NDCNTs show an inferior OER activity with a larger onset potential. For example, for CoO_x_–NDCNTs nanotube catalyst, the current density can reach 38.7 mA cm^−2^ when η = 300 mV, which is 322.5 times than that of the pure g-C_3_N_4_. These results undoubtedly confirm that the effect of CoO_x_ nanoparticles for promoting OER.

The Tafel slope describes the effect of potential or overpotential on the steady-state current density and is an important factor in assessing OER dynamics. At a moderate increase of overpotential, the small Tafel slope will lead to a significantly enhanced OER rate [58]. Tafel plot of the CoO_x_–NDCNTs nanotube shows predominant performance in terms of dynamics compared to the single g-C_3_N_4_. As shown in Figure 8b, the Tafel slope of pure g-C_3_N_4_ is 91 mV dec^−^^1^ and the CoO_x_–NDCNTs nanotube is only 42 mV dec^−1^. The tubular morphology and co-catalytic effect of CoO_x_ may make the catalytic process efficiently.

The turnover frequency (TOF) is an important characterization of the oxygen production performance of the catalyst. As can be seen from Figure 8c, the TOF value of the CoO_x_–NDCNTs nanotube catalyst is 1.2 s^−1^ at η = 300 mV, which shows significant superiority to the single g-C_3_N_4_. These results indicate that the CoO_x_–NDCNTs nanotube catalyst exhibit excellent activity for OER.

Mass activity is also important parameters for studying electrocatalytic oxygen production. As shown in Figure 8d, the mass activity of the CoO_x_–NDCNTs reaches 133 A g^−1^ at η = 300 mV. In contrast, only 0.43 A g^−1^ was obtained for single g-C_3_N_4_ at the same overpotentials, which once again demonstrated the superiority of CoO_x_ nanotube catalyst.

In addition to activity, electrochemical stability is another important index for evaluating electrocatalysts. Long-term cyclic voltammetry of CoO_x_-NDCNTs nanotube was carried out. As shown in Figure 8e, the anodic current increases significantly after 5 CV cycles and reaches a maximum after 100 cycles. This is very common for OER catalysts due to the large accumulation of active high-valence species during the activation process [59,60]. With the cycle further proceeding, a attenuation of the current can be observed. After 1000 CV cycles, the current starts to be little lower than the initial cycle. 

## 4. Conclusions

In conclusion, a new method to synthesize Co(O)_x_–NDCNTs composites by using metal-melamine mixtures as precursor was successfully achieved. CoO_x_–NDCNTs show the good property for the electrocatalytic oxygen production. Co–NDCNTs catalyst was obtained by H_2_-pretreating CoO_x_–NDCNTs catalyst, and the Co–NDCNTs exhibit a strong absorption in the visible range and shows highly photocatalytic activity for H_2_ production. The good catalytic activity should be attributed to the tubular morphology and co-catalytic effect of CoO_x_ or Co nanoparticle. The results imply that supramolecular preorganization method may be promising for preparing two-dimensional N-doped carbon nanotube with metal particles in it. The work can encourage further synthesizing new morphology and noble-metal-free catalysts.

## Supplementary Materials

The following are available online at https://www.mdpi.com/2073-4360/11/11/1836/s1, Figure S1: The picture of CoO_x_-NDCNTs sample; Table S1: The elemental analysis of different samples; Table S2: EQE value calculated at different wavelengths.
