# Supplementary material for: Co(O)x Particles in Polymeric N-Doped Carbon Nanotube Applied for Photocatalytic H2 or Electrocatalytic O2 Evolution
Source: Polymers (Basel). 2019 Nov 7;11(11):1836. doi: 10.3390/polym11111836 (PMC6918141; doi:10.3390/polym11111836)
Supplement: Supplementary file 1 [file polymers-11-01836-s001.pdf]

polymers

MDPI

Article

$Co(O)_x$ Particles in Polymeric N-doped Carbon Nanotube Applied for Photocatalytic H_2 or Electrocatalytic O_2 Evolution

Xiaoxue Zheng †, Ke Liu †, Lichao Chen, Hengxiao He, Lusheng Chen * and Chuanzhi Sun *

College of Chemistry, Chemical Engineering and Materials Science, Collaborative Innovation Center of Functionalized Probes for Chemical Imaging in Universities of Shandong, Shandong Provincial Key Laboratory of Clean Production of Fine Chemicals, Institute of Materials and Clean Energy, Shandong Normal University, Jinan 250014, China

† These two authors contributed equally to this work.

* Correspondence: suncz@sdnu.edu.cn

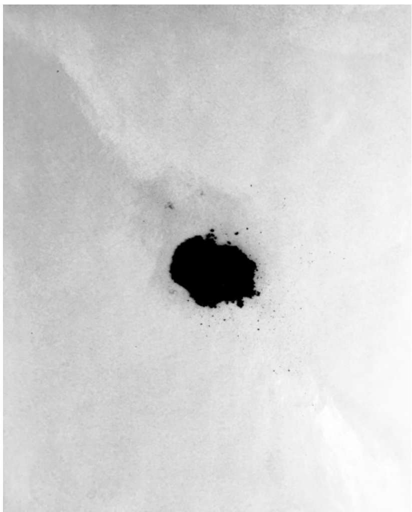

Figure S1. The picture of CoO_x-NDCNTs sample.

Table S1. The elemental analysis of different samples.

Samples	C (%)	N (%)
g-C_3N_4	34.14	60.04
CoO_x-NDCNTs	78.75	17.55
Co-NDCNTs	92.66	6.56

The external quantum efficiency (EQE) test

The external quantum efficiency (EQE) tests were performed in a 20 ml quartz bottle with rubber septum. 10 mg sample was dispersed in aqueous solution (10 ml) containing 20 vol% TEOA. This system was evacuated and bubbled Ar for 10 min to remove the residual gas. Then, the photocatalytic system was irradiated with 300 W Xe arc lamp with a set of band-pass filters (= 405 nm, 420 nm, 450 nm, 500 nm and 650 nm) under magnetic stirring, the irradiation intensities were determined by a NOVA II laser power meter (Ophir Photonics).

Table S2. EQE value calculated at different wavelengths.

**Wavelength**	**H_2 Evolved (μmol)**	**Light Intensity (W)**	**EQE**
405 nm	51.47	0.090	1.83%
420 nm	35.91	0.087	1.28%
450 nm	23.06	0.085	0.78%
500 nm	14.87	0.082	0.47%
650 nm	0.09	—	—
